# Workplace and non-workplace loneliness: a cross-sectional comparative study on risk factors and impacts on absenteeism and mental health among employees in Spain

**DOI:** 10.1007/s00127-025-02899-z

**Published:** 2025-04-24

**Authors:** Joan Domènech-Abella, Jordi Mundó, Josep Maria Haro, Carles Muntaner

**Affiliations:** 1 Centro de Investigación Biomédica en Red de Salud Mental (CIBERSAM), Instituto de Salud Carlos III, Madrid, Spain; 2 Institut de Recerca Sant Joan de Déu, Esplugues de Llobregat, Barcelona, Spain; 3 Department of Sociology, Universitat de Barcelona, Barcelona, Spain; 4 Department of Psychiatry, Universitat de Barcelona, Barcelona, Spain; 5 Dalla Lana School of Public Health, University of Toronto, Toronto, Canada

**Keywords:** Workplace loneliness, Loneliness, Labor precariousness, Absenteeism, Mental health.

## Abstract

**Purpose:**

The aim of this study is to (1) evaluate prevalences and concordance between workplace and non-workplace loneliness, (2) compare sociodemographic risk factors between workplace and non-workplace loneliness, (3) compare working conditions-related risk factors between the two contexts of loneliness, and (4) compare the impact of workplace and non-workplace loneliness on absenteeism, depression, anxiety and substance use disorder.

**Methods:**

A sample of the employee residing in Spain (*n* = 5400) was surveyed using computer-assisted web interviews (CAWI) during August and September 2024. Logistic regression models were constructed to compare the effects of risk factors for workplace and non-workplace loneliness (including sociodemographic factors, and factors related to working conditions), as well as the association of workplace and non-workplace loneliness on absenteeism, and symptoms of depression, anxiety, and substance use disorder.

**Results:**

Among active workers, 40.7% report experiencing workplace loneliness, while 42.0% report non-workplace loneliness. The level of concordance between both types of loneliness is low (Kappa = 0.36). Both types are more prevalent among younger and immigrant workers. Other sociodemographic risk factors (being female, non-married, and non-heterosexual) were significantly associated with non-workplace loneliness. Meanwhile, risk factors related to working conditions -particularly working under stress and labor precariousness- were associated with both types of loneliness, which showed an independent impact on absenteeism, depression, anxiety, and substance use disorder.

**Conclusion:**

Most of the social determinants of workplace loneliness are rooted in the work environment, indicating that effective interventions should focus on addressing labor conditions and precariousness to improve both workplace and non-workplace loneliness and their impacts on absenteeism and mental health.

**Supplementary Information:**

The online version contains supplementary material available at 10.1007/s00127-025-02899-z.

## Introduction

Loneliness is a universal human experience that transcends cultural and socioeconomic boundaries. It is defined as the subjective feeling of isolation or lack of meaningful social connections, even when one is surrounded by others [1]. Loneliness is distinct from social isolation, which refers to an objective lack of social contact. Instead, loneliness arises from a perceived discrepancy between the relationships an individual desires and those they actually experience [2]. It can manifest in various aspects of life, including personal and professional contexts. Although most of studies on loneliness are focused on general loneliness without the specification on any context [3], the study of workplace loneliness has garnered increased attention in recent years and researchers explore how loneliness in professional settings affects labor satisfaction, productivity and mental health of workers [4–5]. Workplace loneliness specifically refers to the emotional disconnect felt by employees who perceive an absence of meaningful relationships at work, often arising from limited social interaction or lack of support from colleagues and supervisors [6–7].

In 2024, the labour force in Spain constituted approximately 51.1% of the total population. Within the labour force, 75.5% were employees, 13% were employers, and 11.5% were unemployed [8]. There is very little information on the prevalence of loneliness among employees in Spain. According to various studies from other countries, workplace loneliness has prevalence rates ranging from 20% in the UK [9] to 62% in the United States [10]. According to the latest study conducted in Spain on loneliness in the general population [11], the prevalence of loneliness was 20%, with a lower prevalence observed among employed participants, including both employees and employers. In addition to contextual and methodological factors, these differences in prevalences are explained by different measures to define the population experiencing loneliness, each considering different frequences and dimensions of loneliness such as experiencing a lack of companionship, feeling left out, and sensing isolation from others [12]. However, despite the differences in prevalence, the risk factors and consequences of loneliness are similar regardless of the measure used, according to previous research on general loneliness [13] and workplace loneliness [14].

Research shows that risk factors for general loneliness include living alone, lacking a partner, having a limited or low-quality social network, unemployment, facing financial difficulties, being an immigrant, and being part of sexual minorities [15–16]. Demographic aspects such as age and gender also play a significant role, with women often reporting higher levels of loneliness, and, particularly since the pandemic, younger adults experiencing increased loneliness [17–18]. Studies on workplace loneliness frequently emphasize the effects of organizational structures that restrict social interaction, such as remote work or isolated departments, along with factors like high levels of work-related pressure and precarious employment, defined by job instability and insecurity, low wages, a lack of benefits, and the absence of legal protection. It also exposes workers to hazardous working conditions and vulnerability to labor abuse, limiting workers’ control over their employment [5, 14, 19].

The detrimental effects of loneliness on mental health are well-documented. Both workplace and general loneliness are linked to increased risks of mental disorders, including depression, anxiety, and substance use disorders [14, 20]. The consequences of workplace loneliness extend beyond the mental health of employees; they also affect organizational outcomes. Employees who feel disconnected from their colleagues or unsupported by their supervisors are more likely to disengage from their work responsibilities, leading to increased absenteeism, reduced productivity, and employee turnover [4, 5, 7]. General loneliness can similarly affect these factors, as individuals who struggle with social isolation may experience greater stress and mental health challenges, leading to more frequent absences from work and less productivity [21]. According to the National Observatory of Unwanted Loneliness in Spain, the annual cost of unwanted loneliness is estimated to be 14 billion euros, which represents approximately 1.17% of the country’s annual gross domestic product [22].

Despite the growing body of research on loneliness, few studies have directly compared workplace and non-workplace loneliness, particularly in terms of their prevalence, agreement with each other, risk factors, and consequences. The present study, with a representative sample of 5400 employees’ residents in Spain aims to (1) evaluate prevalence and concordance between workplace and non-workplace loneliness, (2) compare sociodemographic risk factors between workplace and non-workplace loneliness, (3) compare working conditions-related risk factors between the two contexts of loneliness, and (4) compare the impact of workplace and non-workplace loneliness on absenteeism, depression, anxiety and substance use disorder.

## Materials and methods

### Study design

This is a cross-sectional study with a sample of 5,400 employees residing in Spain, surveyed through the CAWI (Computer-Assisted Web Interviewing) method during August and September 2024. Respondents were selected from a panel managed by IPSOS, which includes 57,093 individuals. The IPSOS panel recruitment and quality process focuses on ensuring accurate population representation rather than simply increasing panel size. Ipsos recruits 7,000 to 10,000 new members each month through vetted vendors and direct partnerships, ensuring panel stability. Strict quality controls are applied at every stage, from panelist registration to survey completion, including multi-factor authentication, bot detection, and fraud prevention measures. For the study sample, we established categories for gender, age, autonomous community, and municipality size to ensure that the sample accurately reflects the distribution of employees in Spain, according to data from the National Statistics Institute [8].

### Measurements

#### Mental disorders symptoms

The two-item Patient Health Questionnaire (PHQ-2) [23] and the two-item Generalized Anxiety Disorders (GAD-2) [24] scales were employed to measure depression and anxiety, respectively, with both scales referring to the past two weeks. Each scale consists of two items: the PHQ-2 includes “Little interest or pleasure in doing things” and “Feeling down, depressed, or hopeless,” while the GAD-2 comprises “Feeling nervous, anxious, or on edge” and “Not being able to stop or control worrying.” Participants respond using the following categories: “Not at all,” “Several days,” “More than half the days,” and “Nearly every day.” The results from both scales range from 0 to 6, with scores of 3 or higher considered compatible with diagnoses of major depressive disorder (MDD) or generalized anxiety disorder (GAD), respectively [23–24].

The CAGE Questionnaire Adapted to Include Drugs (CAGE-AID) [25] was used to assess substance use disorder during the previous month. It consists of four questions with ‘yes’ or ‘no’ answers: (1) “Have you ever felt you ought to cut down on your drinking or drug use?”; (2) “Have people annoyed you by criticizing your drinking or drug use?”; (3) “Have you ever felt bad or guilty about your drinking or drug use?”; and (4) “Have you ever had a drink or used drugs first thing in the morning to steady your nerves or get rid of a hangover?” Answering ‘yes’ to two or more questions determines substance use disorder [25].

#### Loneliness

Loneliness was evaluated through a direct question, “How frequently do you experience loneliness?” as well as through the three-item University of California, Los Angeles (UCLA) Loneliness Scale, which is recognized for its satisfactory reliability and demonstrated concurrent and discriminant validity [12]. This scale includes the following inquiries: “How frequently do you experience a lack of companionship?“; “How often do you feel left out?“; and “How frequently do you sense isolation from others?” Participants rated each question on a 3-point scale (1 = rarely; 2 = sometimes; 3 = frequently). The cumulative score can range from 3 to 9, with higher scores indicating greater feelings of loneliness. In this study, loneliness was defined as reporting “sometimes” or “frequently” feeling lonely according to the direct question or achieving a cutoff score of ≥ 6 [13] on the UCLA loneliness scale [12]. In both cases, loneliness was assessed, separately, in workplace and non-workplace contexts. Primary analyses were conducted using the direct question. In the supplementary material, analyses were replicated using the cutoff score of ≥ 6 from the UCLA scale.

#### Sociodemographic variables

Sociodemographic information included gender (male, female, and other), age groups (18–29 years, 30–39 years, 40–49 years, 50–59 years, and 60–65 years), marital status (married, including civil partnerships; never married; separated, including divorced and widowed), nationality (Spanish or non-Spanish), sexual orientation (heterosexual or non-heterosexual), and occupation level (directors, including managers; professionals, including scientists and intellectuals; other non-manual roles, comprising technicians, middle-level professionals, and administrative staff; skilled manual workers, including commerce workers, tradespeople, artisans, and qualified workers; unskilled manual workers, including machine operators and elementary occupations; and military personnel) [26].

#### Working conditions-related variables

Working conditions-related variables included manager status (yes, no), which indicates whether the individual has employees under their supervision; length of service (less than 1 year, 1 to 3 years, more than 3 years); teleworking days (none, less than half, half or more); working under pressure (no, more or less, yes); sick leave days in the last year (5 or fewer days / more than 5 days); and frequent communication with colleagues (yes, more or less, no), which aims to reflect social interactions in the workplace, assessing to what extent work is carried out in isolation or through collaboration.

The Employment Precariousness Scale for Europe (EPRES-E) was utilized to assess labor precariousness. This scale comprises six domains: temporariness (contract duration), disempowerment (the level of negotiation regarding employment conditions), vulnerability (defenselessness against workplace authoritarianism), salary, workplace rights (reconciliation with daily needs), and unpredictability (the predictability of work schedules). Each dimension was measured using ordinal scales, which were recoded into subscales ranging from 0 to 100, where higher values indicate more precarious situations. The total score was derived as a simple average of the six subscales, resulting in a final score ranging from 0 to 100 [27–28].

### Statistical analysis

Descriptive analyses were reported overall and for subpopulations with workplace loneliness and non-workplace loneliness. Proportions and frequencies for categorical variables and means and standard deviation of continuous variables were reported. Differences between population with and without workplace loneliness as well as with and without non-workplace loneliness were measured through Chi-squared test for categorical variables and *t*-test for continuous variables. Additionally, a Kappa statistic was calculated to assess the level of agreement between workplace loneliness and non-workplace loneliness.

Two separate adjusted logistic regression models were developed to investigate sociodemographic risk factors for both workplace and non-workplace loneliness. Both models incorporated all sociodemographic variables simultaneously (gender, age groups, nationality, marital status, sexual orientation and occupation level). Odds ratio with 95% confidence interval were reported.

Similarly, two separate adjusted logistic regression models were constructed to investigate working conditions-related risk factors for both workplace and non-workplace loneliness. Both models incorporated all working condition-related variables (manager/supervisor, length of service, teleworking days, working under pressure, frequent communication, and labor precariousness) as well gender and age groups.

Unlike categorical variables, the interpretation of odds ratios for continuous variables is more complex. Since precariousness was the only continuous variable, to clarify the association between precariousness and workplace and non-workplace loneliness, estimated probabilities with 95% confidence interval for both contexts of loneliness were calculated. These probabilities were determined using the “margins” command in Stata [29], based on the adjusted logistic regression models for workplace loneliness and non-workplace loneliness. Control variables were centered to their means, considering the sample proportions.

To evaluate the impact of workplace and non-workplace loneliness on absenteeism (more than five days off in the last year), depression, anxiety and substance use disorder, additional logistic regression models were constructed. Firstly, we included as independent variable workplace and non-workplace loneliness separately, and, finally, we included both variables simultaneously. In all cases, gender and age groups were included as covariates. Odds ratio with 95% confidence interval were reported.

While these preliminary analyses were conducted using loneliness defined by a direct question as the dependent variable, sensitivity analyses were also performed replicating all analyses using the definition of loneliness based on a cutoff of ≥ 6 on the three-item UCLA loneliness scale. All reported p-values were based on a two-sided test, where the level of statistical significance was set at *p* < 0.05. Stata version 13 [30] was used to analyze the survey data.

## Results

The characteristics of the study sample and participants reporting either workplace or non-workplace loneliness are presented in Table 1. A total of 40.7% of participants reported workplace loneliness whereas 42.0% of participants reported non-workplace loneliness. The Kappa statistic was 0.36, indicating a fair level of agreement between workplace and non-workplace loneliness [31].

Younger and immigrant workers, as well as those who telework, work under pressure, report maintaining moderate communication with their colleagues, with 1 to 3 years of service and experience high levels of labor precariousness reported loneliness in both contexts more frequently. In addition, women, unmarried participants, non-Spanish individuals, and non-heterosexuals, reported non-workplace loneliness more frequently, while those with employees under their supervision reported workplace loneliness more frequently.

**Table 1:** Characteristics of the study sample

Characteristic	Overall (*n* = 5400)	Workplace loneliness	*p* value^1^	Non-workplace loneliness	*p* value^2^
Gender					
• Male	2753 (51.0)	1107 (40.2)	> 0.05	1035 (37.6)	**< 0.001**
• Female	2631 (48.7)	1081 (41.1)		1226 (46.6)	
• Other	16 (0.3)	8 (50.0)		7 (43.8)	
Age groups					
• 60–65	360 (6.7)	111 (30.8)	**< 0.001**	120 (33.3)	**< 0.001**
• 50–59	1395 (25.8)	526 (37.7)		186 (34.8)	
• 40–49	1598 (62.1)	649 (40.6)		631 (39.5)	
• 30–39	1319 (24.4)	590 (44.7)		663 (50.3)	
• 18–29	728 (13.5)	320 (44.0)		368 (50.6)	
Marital status					
• Married	3518 (65.2)	1412 (40.1)	> 0.05	1314 (37.4)	**< 0.001**
• Never married	1457 (27.0)	626 (43.0)		755 (51.8)	
• Separated	392 (7.2)	147 (37.5)		181 (46.2)	
• Widowed	33 (0.6)	11 (33.3)		18 (54.6)	
Nationality					
• Non-Spanish	216 (4.0)	109 (50.5)	**< 0.01**	111 (51.4)	**< 0.01**
Sexual orientation					
• Non-heterosexual	609 (11.28)	270 (44.3)	> 0.05	315 (51.7)	**< 0.001**
Occupation					
• Directors	306 (5.7)	121 (39.5)	> 0.05	112 (36.6)	**< 0.05**
• Professionals	522 (9.7)	213 (40.8)		231 (44.3)	
• Another non-manual	3020 (55.9)	1203 (39.8)		1282 (42.5)	
• Skilled manual	886 (16.4)	389 (43.9)		381 (43.0)	
• Unskilled manual	600 (11.1)	247 (41.2)		245 (40.8)	
• Military personnel	66 (1.2)	23 (34.9)		17 (25.8)	
Manager / supervisor					
• Yes	1499 (27.8)	659 (44.0)	**< 0.01**	634 (42.3)	> 0.05
Length of service					
• Less than 1 year	517 (9.6)	210 (40.6)	> 0.05	216 (41.8)	**< 0.001**
• From 3 to 1 year	928 (17.2)	405 (43.6)		444 (47.8)	
• More than 3 years	3955 (73.2)	1581 (40.0)		1608 (40.7)	
Teleworking days					
• None	3541 (65.6)	1380 (39.0)	**< 0.01**	1404 (39.7)	**< 0.001**
• Less than half	943 (17.5)	411 (43.6)		462 (49.0)	
• Half or more	916 (17.0)	405 (44.2)		402 (43.9)	
Working under pressure					
• No	2536 (47.0)	665 (26.2)	**< 0.001**	802 (31.6)	**< 0.001**
• More or less	1838 (34.0)	866 (47.1)		909 (49.5)	
• Yes	1026 (19.0)	665 (64.8)		557 (54.3)	
Frequent communication					
• Yes	3229 (59.8)	1225 (37.9)	**< 0.001**	1287 (39.9)	**< 0.001**
• More or less	1709 (31.7)	787 (46.1)		799 (46.8)	
• No	462 (8.6)	184 (39.8)		182 (39.4)	
Labor precariousness (0-100)	27.5 (14.4) *	32.7 (14.6) *	**< 0.001**	30.6 (14.4) *	**< 0.001**
**Outcomes**:					
> 5 days of sick leave last year	1042 (19.3)	559 (53.7)	**< 0.001**	559 (53.7)	**< 0.001**
Depression	1035 (19.2)	685 (66.2)	**< 0.001**	693 (67.0)	**< 0.001**
Anxiety	988 (18.3)	676 (68.4)	**< 0.001**	670 (67.8)	**< 0.001**
Substance use disorder	512 (9.6)	314 (61.3)	**< 0.001**	324 (63.3)	**< 0.001**
Workplace loneliness	2196 (40.7)	-	-	1390 (63.3)	**< 0.001** K = 0.36
Non-workplace loneliness	2268 (42.0)	1390 (61.3)	**-**	-	

Note = Frequencies with percentages and (*) means with standard deviations are reported. P-value for differences between (^1^) populations with and without workplace loneliness and (^2^) populations with and without non-workplace loneliness. The degree of agreement between workplace and non-workplace loneliness was measured using kappa (K)

Tables 2 and 3 present the outcomes of adjusted logistic regression models evaluating the relationship of key variables with workplace and non-workplace loneliness. Statistically significant associations indicated that the significant relationships identified in the bivariate analyses remain after adjusting the models. The only noteworthy change is that individuals with more than three years of seniority experience both workplace and non-workplace loneliness more frequently compared to those who joined in the last year.

**Table 2:** Adjusted logistic regression models of sociodemographic factors related with workplace and non-workplace loneliness

Characteristic	Workplace loneliness	Non-workplace loneliness
Gender		
• Male	Ref.	Ref.
• Female	1.00 (0.89, 1.12)	1.29 (1.15, 1.45)***
• Other	1.37 (0.51, 3.69)	0.83 (0.30, 2.29)
Age groups		
• 60–65	Ref.	Ref.
• 50–59	1.35 (1.05, 1.74)*	1.08 (0.84, 1.39)
• 40–49	1.52 (1.18, 1.94)**	1.32 (1.03, 1.68)*
• 30–39	1.78 (1.38, 2.29)***	1.86 (1.45, 2.39)***
• 18–29	1.64 (1.24, 2.16)**	1.63 (1.24, 2.16)**
Marital status		
• Married	Ref.	Ref.
• Never married	1.04 (0.91, 1.18)	1.53 (1.34, 1.74)***
• Separated	0.96 (0.77, 1.19)	1.61 (1.30, 1.99)***
• Widowed	0.81 (0.39, 1.71)	2.09 (1.04, 4.21)*
Nationality		
• Spanish	Ref.	Ref.
• Non-Spanish	1.45 (1.10, 1.91)**	1.33 (1.00, 1.76)*
Sexual orientation		
• Heterosexual	Ref.	Ref.
• Non-heterosexual	1.11 (0.93, 1.32)	1.39 (1.17, 1.66)***
Occupation		
• Directors	Ref.	Ref.
• Professionals	1.04 (0.77, 1.38)	1.25 (0.93, 1.69)
• Another non-manual	1.03 (0.81, 1.31)	1.26 (0.99, 1.62)
• Skilled manual	1.20 (0.92, 1.56)	1.30 (0.99, 1.71)
• Unskilled manual	1.10 (0.83, 1.47)	1.25 (0.93, 1.67)
• Military personnel	0.86 (0.49, 1.50)	0.70 (0.38, 1.29)

Note = Odds ratio with 95% confidence interval are reported

Ref.=category of reference

**Table 3:** Adjusted logistic regression models of working conditions-related factors associated with workplace and non-workplace loneliness

Characteristic	Workplace loneliness	Non-workplace loneliness
Manager / supervisor		
• No	Ref.	Ref.
• Yes	1.18 (1.02, 1.35)*	0.96 (0.84, 1.10)
Length of service		
• Less than 1 year	Ref.	Ref.
• From 3 to 1 year	1.24 (0.98, 1.58)	1.36 (1.08, 1.71)**
• More than 3 years	1.46 (1.17, 1.83)**	1.39 (1.12, 1.71)**
Teleworking days		
• None	Ref.	Ref.
• Less than half	1.22 (1.03, 1.43)*	1.40 (1.20, 1.64)***
• Half or more	1.36 (1.16, 1.60)***	1.19 (1.02, 1.39)*
Working under pressure		
• No	Ref.	Ref.
• More or less	2.02 (1.76, 2.31)***	1.81 (1.59, 2.07)***
• Yes	3.60 (3.03, 4.27)***	1.96 (1.66, 2.31)***
Frequent communication		
• Yes	Ref.	Ref.
• More or less	1.46 (1.17, 1.83)**	1.31 (1.15, 1.48)***
• No	1.24 (0.98, 1.58)	1.10 (0.89, 1.35)
Labor precariousness (0-100)	1.04 (1.04, 1.05)***	1.02 (1.02, 1.03)***

Note = Odds ratio with 95% confidence interval are reported. Age and gender were included as covariates

Ref.=category of reference

**Fig. 1:**
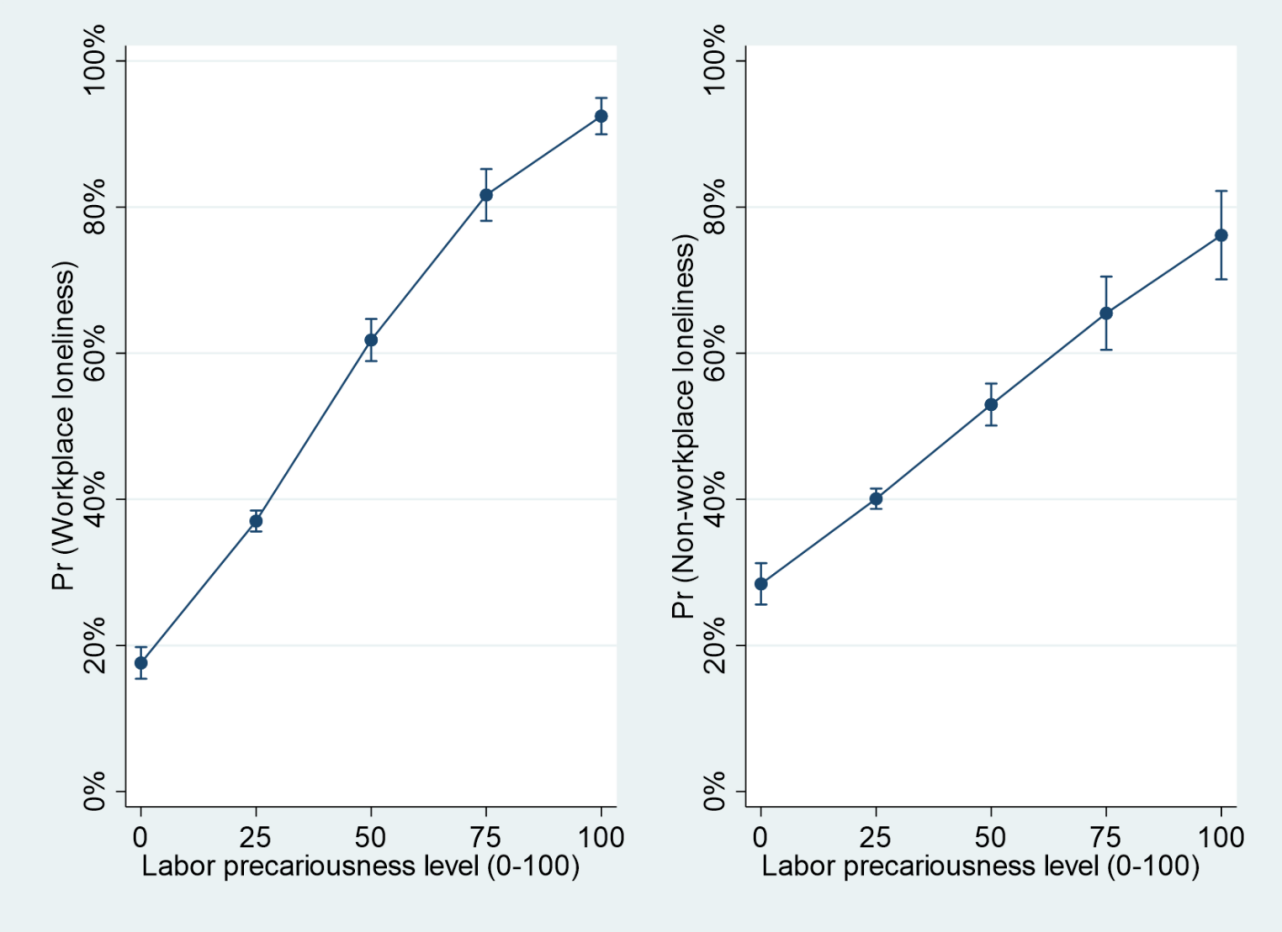
Estimated probabilities of workplace and non-workplace loneliness according to labor precariousness level. Note = estimated probabilities and 95% confidence intervals from Model 2 of Table 3, with covariates centered at the mean, are reported

The estimated probabilities for workplace and non-workplace loneliness, depending on the labor precariousness level, are represented in Fig. 1. In both cases, higher levels of precariousness were associated with higher probabilities of loneliness. Depending on the level of precariousness, the probability of workplace loneliness ranged from 0.18 (95% CI 0.15, 0.20) to 0.92 (95% CI 0.90, 0.95), while non-workplace loneliness ranged from 0.28 (95% CI 0.26, 0.31) to 0.76 (95% CI 0.70, 0.82).

Table 4 presents the outcomes of adjusted logistic regression models evaluating the relationship of workplace and non-workplace loneliness with absenteeism, depression, anxiety and substance use disorder. In all cases, loneliness significantly increased the odds for absenteeism, depression, anxiety and substance use disorder before and after adjusting the models for workplace and non-workplace loneliness simultaneously.

**Table 4:** Logistic regression models of workplace and non-workplace loneliness associated with absenteeism, and depressive, anxiety and substance use disorder symptoms

Characteristic	Absenteeism	Depression	Anxiety	Substance use
	Model 1	Model 1	Model 1	Model 1
Workplace loneliness				
• No	Ref.	Ref.	Ref.	Ref.
• Yes	1.91 (1.67, 2.19)***	3.61 (3.12, 4.17)***	4.05 (3.49, 4.71)***	2.49 (2.06, 3.01)***
Non-workplace loneliness				
• No	Ref.	Ref.	Ref.	Ref.
• Yes	1.76 (1.53, 2.02)***	3.36 (2.90, 3.88)***	3.44 (2.97, 4.00)***	2.60 (2.15, 3.15)***
	**Model 2**	**Model 2**	**Model 2**	**Model 2**
Workplace loneliness				
• No	Ref.	Ref.	Ref.	Ref.
• Yes	1.67 (1.45, 1.94)***	2.72 (2.34, 3.17)***	3.07 (2.62, 3.60)***	1.94 (1.59, 2.37)***
Non-workplace loneliness				
• No	Ref.	Ref.	Ref.	Ref.
• Yes	1.47 (1.27, 1.70)***	2.43 (2.08, 2.83)***	2.41 (2.06, 2.82)***	2.06 (1.68, 2.52)***

Note = Odds ratio with 95% confidence interval are reported. In the Model 1, each variable shown is analysed separately, while in the Model 2, the variables are adjusted for each other. In all cases, sex and age are included as covariates. Ref.=category of reference

The supplementary materials include sensitivity analyses that replicate these assessments using the UCLA scale to define loneliness. Table S1 indicates a prevalence of 26.7% for workplace loneliness and 26.9% for non-workplace loneliness. Both types of loneliness demonstrate moderate agreement with each other (K = 0.44). Table S2, Table S3, and Table S4, along with Figure S1, reveal statistically significant associations consistent with those presented earlier.

## Discussion

To the best of our knowledge, this is the first study to compare workplace and non-workplace loneliness in a representative sample of employees, examining their risk factors and impacts on absenteeism and mental health. According to our results, the level of concordance between the two types of loneliness is low (k = 0.36). Both types of loneliness are more prevalent among younger workers and immigrants. Other sociodemographic risk factors, such as being female, non-married, and non-heterosexual, were significantly associated with non-workplace loneliness, whereas most of working conditions-related factors were associated with both types of loneliness, which independently impacted absenteeism, depression, anxiety, and substance use disorder.

Among active workers, 40.7% reported experiencing workplace loneliness when responding to the direct question, while 42.0% reported non-workplace loneliness. In the sensitivity analysis, using a cutoff point of 6 on the 3-item UCLA loneliness scale, the prevalence for workplace loneliness was 26.7% and for non-workplace loneliness was 26.9%.

Although comparisons are limited due to differences in the frequency of loneliness measured (e.g., “sometimes” + often” vs. “often”), the workplace loneliness prevalence from the direct question (40.7%) falls between the rates reported in UK studies (20%) [9] and those from the United States (62%) [10], whereas the prevalence measured using the 3-item UCLA scale aligns with previous research on general loneliness in Spain (20%) [11]. Although there is a lack of information about the agreement between general loneliness and workplace and non-workplace loneliness, our results suggest that the employee population experiences slightly higher loneliness prevalence compared to the general population, which may be due to increased exposure to work-related risk factors, as well as the fact that the employee population excludes older adults, who tend to report lower levels of loneliness, according to recent studies [17–18].

In line with findings from studies on work-to-family spillover effects related to negative workplace experiences, which highlight the need for employees to establish boundaries between their work and family lives [32–33], our results indicate that labor conditions and workplace loneliness are associated with non-workplace loneliness. However, the agreement between these two types of loneliness is weak, suggesting that, largely, one type does not imply the other.

While workplace and non-workplace loneliness share some common risk factors linked to general loneliness [15], such as being an immigrant or being younger adult, non-workplace loneliness is also associated with being female, unmarried, and non-heterosexual. In contrast, workplace loneliness is more strongly linked to work-related risk factors, most of which are also associated with non-workplace loneliness.

According to our results, having subordinates is a risk factor exclusive to workplace loneliness. Previous studies have also noted this, finding that workers with subordinates more frequently report mental health problems. This has been attributed to a ‘contradictory class position,’ where workers are often pressured by their superiors while facing conflicts with their subordinates [34–35].

Teleworking, maintaining moderate or low communication with colleagues, having more than three years of tenure at the company, working under stress and labor precariousness are risk factors shared by workplace and non-workplace loneliness. While the impact of limited relationships with colleagues and teleworking on loneliness is supported by previous studies [14, 19, 36], the finding that more tenured workers experience loneliness more frequently contrasts with earlier results [7]. However, this discrepancy is shown exclusively in adjusted models and can be attributed to the influence of adjustment variables, which account for additional factors affecting the relationship between tenure and loneliness [37].

Working under stress and labor precariousness were the factors more strongly associated with both types of loneliness and, particularly, with workplace loneliness. Working under pressure increases the likelihood of experiencing workplace loneliness by up to 3.6 times. Although limited information exists on these associations, previous studies have highlighted the impact of stress on loneliness and mental health [20, 38]. In addition, labor precariousness is an almost perfect determinant of the prevalence of workplace loneliness. In line with this, previous studies using data from several European countries demonstrated the significant impact of precarious work on mental health [39–41].

The impact of loneliness on symptoms of depression, anxiety, and substance use disorders [14, 20] as well as on absenteeism [4, 7] has been widely reported. In this context, our results confirm that both workplace and non-workplace loneliness are assocaiated with work conditions-related risk factors and have an independent effect on absenteeism and mental health.

### Strengths and limitations

This study’s strengths lie in its large and diverse sample size, which effectively stratifies across all key socio-demographic groups, including various occupational categories and leadership roles. However, these findings should be interpreted with several limitations in mind. First, our data rely on self-reported measures, which may introduce reporting or recall bias. Nevertheless, the short and clearly defined recall periods in our study help mitigate this risk. Second, the cross-sectional nature of the study restricts the exploration of causal relationships. We have proposed loneliness as a risk factor for mental disorders based on previous evidence [42], although the relationship could also occur in the opposite direction. Despite this, we accounted for key confounding variables and utilized a variety of validated scales to measure the study variables. Third, while our sample is representative of active workers in Spain, caution is warranted when generalizing these results to other countries due to contextual differences Finally, the lack of a commonly accepted threshold to define loneliness may lead to variability in results. To address this, we applied two loneliness measures and used previously established cut-off points. Our results are consistent with a prior study, which demonstrated that the behavior of risk factors is similar in both cases [13]. Future research in varied regions and with fewer limitations should aim to investigate the causality of the relationships identified in this study.

## Conclusions

Our results suggest that, like non-workplace loneliness, workplace loneliness is a prevalent condition among workers. Working conditions-related factors are associated with both forms of loneliness, particularly workplace loneliness. Therefore, interventions aimed at improving working conditions -such as reducing pressure to meet performance targets and addressing job precariousness through measures like longer contract duration, greater negotiation power over employment terms, protection against workplace authoritarianism, fair salaries, strengthened workplace rights, predictable work schedules, fewer supervision layers, or legislation that empowers workers- could significantly reduce both workplace and non-workplace loneliness, decrease absenteeism, and improve employees’ mental health.

## Electronic supplementary material

Below is the link to the electronic supplementary material.

Supplementary Material 1

## Data Availability

Data from the Edad con Salud (EcS) are available for use for specific research questions, provided that an agreement is made up. Research proposals should be submitted to the EcS Steering Group, using a standard analysis proposal form that can be obtained from the EcS website: (http://edadconsalud.com/contacto/?lang=en). Files with data published in this publication are freely available for replication purposes and can be obtained using the same analysis proposal form. The EcS Steering Group will review all requests for data to ensure that proposals for the use of EcS data do not violate privacy regulations and are in keeping with informed consent that is provided by all EcS participants.
