# Supplementary material for: Workplace and non-workplace loneliness: a cross-sectional comparative study on risk factors and impacts on absenteeism and mental health among employees in Spain
Source: Soc Psychiatry Psychiatr Epidemiol. 2025 Apr 24;60(10):2289–99. doi: 10.1007/s00127-025-02899-z (PMC12449428; doi:10.1007/s00127-025-02899-z)
Supplement: Supplementary file 1 — Supplementary Material 1 [file 127_2025_2899_MOESM1_ESM.docx]

**Supplementary material**

**Supplementary Table S1**. Characteristics of the study sample using UCLA scale.

**Supplementary Table S2**. Adjusted logistic regression models of sociodemographic factors related with workplace and non-workplace loneliness measured by UCLA scale.

**Supplementary Table S3.** Adjusted logistic regression models of working conditions-related factors associated with workplace and non-workplace loneliness measured by UCLA scale.

**Supplementary Table S4.** Logistic regression models of workplace and non-workplace loneliness measured by UCLA scale associated with absenteeism, and depressive, anxiety and substance use disorder symptoms.

**Supplementary Figure S1.** Estimated probabilities of workplace and non-workplace loneliness measured by UCLA scale according to labor precariousness level.

NOTE: The sensitivity analyses indicate that, apart from workplace (26.7%) and non-workplace (26.9%) loneliness prevalences and being an immigrant worker, which was not found as risk factor, remaining results have remained largely unaffected by the loneliness measure. This is evident from the comparable distribution of outcome variables (compare **Table 1** with **Table S1**). The identified risk factors for workplace and non-workplace loneliness exhibit similar magnitudes (compare **Table 2**, **Table 3** and **Figure 1** with **Table S2, Table S3** and **Figure S1**), and the association of workplace and non-workplace loneliness with absenteeism and mental health consistently demonstrates strong correlations (compare **Table 4** with **Table S4**).

**Table S1.** Characteristics of the study sample using UCLA scale.

| **Characteristic** | **Overall**  **(n=5400)** | **Workplace loneliness** | ***p* value^1^** | **Non-workplace loneliness** | ***p* value^2^** |
| --- | --- | --- | --- | --- | --- |
| Gender |  |  |  |  |  |
| - Male | 2753 (51.0) | 705 (25.6) | >0.05 | 661 (24.0) | **<0.001** |
| - Female | 2631 (48.7) | 730 (27.8) |  | 783 (29.8) |  |
| - Other | 16 (0.3) | 6 (37.5) |  | 6 (37.5) |  |
| Age groups |  |  |  |  |  |
| - 60-65 | 360 (6.7) | 65 (18.1) | **<0.001** | 64 (17.8) | **<0.001** |
| - 50-59 | 1395 (25.8) | 330 (23.7) |  | 291 (20.9) |  |
| - 40-49 | 1598 (62.1) | 422 (26.4) |  | 407 (25.5) |  |
| - 30-39 | 1319 (24.4) | 395 (30.0) |  | 440 (33.4) |  |
| - 18-29 | 728 (13.5) | 229 (31.5) |  | 248 (34.1) |  |
| Marital status |  |  |  |  |  |
| - Married | 3518 (65.2) | 935 (26.6) | >0.05 | 844 (24.0) | **<0.001** |
| - Never married | 1457 (27.0) | 402 (27.6) |  | 482 (33.1) |  |
| - Separated | 392 (7.2) | 98 (25.0) |  | 113 (28.8) |  |
| - Widowed | 33 (0.6) | 6 (18.2) |  | 11 (33.3) |  |
| Nationality |  |  |  |  |  |
| - Non-Spanish | 216 (4.0) | 70 (32.4) | >0.05 | 71 (32.9) | **<0.05** |
| Sexual orientation |  |  |  |  |  |
| - Non-heterosexual | 609 (11.28) | 184 (30.2) | **<0.05** | 207 (34.0) | **<0.001** |
| Occupation |  |  |  |  |  |
| - Directors | 306 (5.7) | 73 (23.9) | >0.05 | 79 (25.8) | >0.05 |
| - Professionals | 522 (9.7) | 141 (27.0) |  | 152 (29.1) |  |
| - Another non-manual | 3020 (55.9) | 806 (26.7) |  | 791 (26.2) |  |
| - Skilled manual | 886 (16.4) | 262 (29.6) |  | 258 (29.1) |  |
| - Unskilled manual | 600 (11.1) | 146 (24.3) |  | 160 (26.7) |  |
| - Military personnel | 66 (1.2) | 13 (19.7) |  | 10 (15.2) |  |
| Manager / supervisor |  |  |  |  |  |
| - Yes | 1499 (27.8) | 456 (30.4) | **<0.001** | 436 (29.1) | **<0.05** |
| Length of service |  |  |  |  |  |
| - Less than 1 year | 517 (9.6) | 134 (25.9) | >0.05 | 1036 (26.2) | **<0.05** |
| - From 3 to 1 year | 928 (17.2) | 247 (26.6) |  | 280 (30.2) |  |
| - More than 3 years | 3955 (73.2) | 1060 (26.8) |  | 134 (25.9) |  |
| Teleworking days |  |  |  |  |  |
| - None | 3541 (65.6) | 881 (24.9) | **<0.01** | 878 (24.8) | **<0.001** |
| - Less than half | 943 (17.5) | 305 (32.3) |  | 316 (33.5) |  |
| - Half or more | 916 (17.0) | 255 (27.8) |  | 256 (28.0) |  |
| Working under pressure |  |  |  |  |  |
| - No | 2536 (47.0) | 351 (13.8) | **<0.001** | 428 (16.9) | **<0.001** |
| - More or less | 1838 (34.0) | 606 (33.0) |  | 633 (34.4) |  |
| - Yes | 1026 (19.0) | 484 (47.2) |  | 389 (37.9) |  |
| Frequent communication |  |  |  |  |  |
| - Yes | 3229 (59.8) | 767 (23.8) | **<0.001** | 797 (24.7) | **<0.001** |
| - More or less | 1709 (31.7) | 570 (33.4) |  | 534 (31.3) |  |
| - No | 462 (8.6) | 104 (22.5) |  | 119 (25.8) |  |
| Labor precariousness (0-100) | 27.5 (14.4) * | 34.5 (14.4) * | **<0.001** | 32.2 (14.4) * | **<0.001** |
| **Outcomes:** |  |  |  |  |  |
| >5 days of sick leave last year | 1042 (19.3) | 430 (41.3) | **<0.001** | 390 (37.4) | **<0.001** |
| Depression | 1035 (19.2) | 557 (53.8) | **<0.001** | 560 (54.1) | **<0.001** |
| Anxiety | 988 (18.3) | 541 (54.8) | **<0.001** | 527 (53.3) | **<0.001** |
| Substance use disorder | 512 (9.6) | 243 (47.5) | **<0.001** | 243 (47.5) | **<0.001** |
| Workplace loneliness | 1441 (26.7) | - | - | 849 (58.9) | **<0.001**  K=0.44 |
| Non-workplace loneliness | 1450 (26.9) | 849 (58.6) | **-** | - |  |

Note= Frequencies with percentages and (*) means with standard deviations are reported. P-value for differences between (^1^) populations with and without workplace loneliness and (^2^) populations with and without non-workplace loneliness. The degree of agreement between workplace and non-workplace loneliness was measured using kappa (K).

**Table S2.** Adjusted logistic regression models of sociodemographic factors related with workplace and non-workplace loneliness measured by UCLA scale.

| **Characteristic** | **Workplace loneliness** | **Non-workplace loneliness** |
| --- | --- | --- |
| Gender |  |  |
| - Male | Ref. | Ref. |
| - Female | 1.06 (0.93, 1.20) | 1.20 (1.06, 1.37)** |
| - Other | 1.55 (0.55, 4.33) | 1.33 (0.47, 3.76) |
| Age groups |  |  |
| - 60-65 | Ref. | Ref. |
| - 50-59 | 1.40 (1.04, 1.89)* | 1.23 (0.91, 1.67) |
| - 40-49 | 1.62 (1.21, 2.17)** | 1.60 (1.19, 2.16)** |
| - 30-39 | 1.91 (1.42, 2.58)*** | 2.20 (1.63, 2.97)*** |
| - 18-29 | 2.02 (1.46, 2.79)*** | 2.04 (1.47, 2.82)*** |
| Marital status |  |  |
| - Married | Ref. | Ref. |
| - Never married | 0.92 (0.80, 1.07) | 1.31 (1.13, 1.52)*** |
| - Separated | 1.00 (0.78, 1.28) | 1.48 (1.16, 1.87)** |
| - Widowed | 0.68 (0.28, 1.67) | 1.75 (0.83, 3.68) |
| Nationality |  |  |
| - Spanish | Ref. | Ref. |
| - Non-Spanish | 1.26 (0.94, 1.69) | 1.19 (0.89, 1.61) |
| Sexual orientation |  |  |
| - Heterosexual | Ref. | Ref. |
| - Non-heterosexual | 1.15 (0.95, 1.39) | 1.31 (1.09, 1.58)** |
| Occupation |  |  |
| - Directors | Ref. | Ref. |
| - Professionals | 1.15 (0.83, 1.60) | 1.09 (0.79, 1.50) |
| - Another non-manual | 1.19 (0.90, 1.57) | 1.02 (0.77, 1.34) |
| - Skilled manual | 1.35 (0.99, 1.82) | 1.17 (0.87, 1.58) |
| - Unskilled manual | 1.08 (0.78, 1.49) | 1.10 (0.80, 1.51) |
| - Military personnel | 0.84 (0.43, 1.64) | 0.59 (0.28, 1.22) |

Note= Odds ratio with 95% confidence interval are reported.

Ref.=category of reference

**Table S3.** Adjusted logistic regression models of working conditions-related factors associated with workplace and non-workplace loneliness measured by UCLA scale.

| **Characteristic** | **Workplace loneliness** | **Non-workplace loneliness** |
| --- | --- | --- |
| Manager / supervisor |  |  |
| - No | Ref. | Ref. |
| - Yes | 1.25 (1.07, 1.45)** | 1.11 (0.95, 1.28) |
| Length of service |  |  |
| - Less than 1 year | Ref. | Ref. |
| - From 3 to 1 year | 1.15 (0.88, 1.51) | 1.32 (1.03, 1.72)* |
| - More than 3 years | 1.77 (1.37, 2.27)*** | 1.55 (1.22, 1.97)*** |
| Teleworking days |  |  |
| - None | Ref. | Ref. |
| - Less than half | 1.48 (1.24, 1.77)*** | 1.44 (1.22, 1.71)*** |
| - Half or more | 1.28 (1.07, 1.54)** | 1.19 (1.00, 1.41)* |
| Working under pressure |  |  |
| - No | Ref. | Ref. |
| - More or less | 2.32 (1.99, 2.73)*** | 2.13 (1.83, 2.47)*** |
| - Yes | 3.50 (2.91, 4.21)*** | 2.11 (1.76, 2.53)*** |
| Frequent communication |  |  |
| - Yes | Ref. | Ref. |
| - More or less | 1.66 (1.44, 1.91)*** | 1.35 (1.18, 1.55)*** |
| - No | 1.07 (0.82, 1.38) | 1.22 (0.97, 1.56) |
| Labor precariousness (0-100) | 1.05 (1.04, 1.05)*** | 1.03 (1.02, 1.03)*** |

Note= Odds ratio with 95% confidence interval are reported. Sex and gender were included as covariates.

Ref.=category of reference

|

**Figure S1.** Estimated probabilities of workplace and non-workplace loneliness measured by UCLA scale according to labor precariousness level.

Note= estimated probabilities and 95% confidence intervals from Model 2 of Table 3, with covariates centered at the mean, are reported.

**Table S4.** Logistic regression models of workplace and non-workplace loneliness measured by UCLA scale associated with absenteeism, and depressive, anxiety and substance use disorder symptoms.

| **Characteristic** | **Absenteeism** | **Depression** | **Anxiety** | **Substance use** |
| --- | --- | --- | --- | --- |
|  | **Model 1** | **Model 1** | **Model 1** | **Model 1** |
| Workplace loneliness |  |  |  |  |
| - No | Ref. | Ref. | Ref. | Ref. |
| - Yes | 2.31  (2.00, 1.66)*** | 4.46  (3.86, 5.15)*** | 4.60  (3.96, 5.33)*** | 2.75  (2.28, 3.31)*** |
| Non-workplace loneliness |  |  |  |  |
| - No | Ref. | Ref. | Ref. | Ref. |
| - Yes | 1.83  (1.58, 2.11)*** | 4.32  (3.74, 4.99)*** | 4.02  (3.47, 4.66)*** | 2.72  (2.25, 3.28)*** |
|  | **Model 2** | **Model 2** | **Model 2** | **Model 2** |
| Workplace loneliness |  |  |  |  |
| - No | Ref. | Ref. | Ref. | Ref. |
| - Yes | 2.04  (1.74, 2.39)*** | 2.94  (2.50, 3.45)*** | 3.15  (2.68, 3.71)*** | 2.03  (1.65, 2.51)*** |
| Non-workplace loneliness |  |  |  |  |
| - No | Ref. | Ref. | Ref. | Ref. |
| - Yes | 1.33  (1.13, 1.57)** | 2.77  (2.36, 3.26)*** | 2.50  (2.12, 2.95)*** | 1.98  (1.60, 2.44)*** |

Note= Odds ratio with 95% confidence interval are reported. In the Model 1, each variable shown is analysed separately, while in the Model 2, the variables are adjusted for each other. In all cases, sex and age are included as covariates. Ref.=category of reference
